# Ionic Liquid-Mediated Modulation of Zwitterionic Micelles and Their Catalytic Performance in the Decarboxylation of 6‑NBIC

**DOI:** 10.1021/acsomega.6c00719

**Published:** 2026-04-27

**Authors:** Paulo F. A. Costa, Victória R. Soares, Yasmin S. Gomes, Hugo Gallardo, Frank H. Quina, Adriana P. Gerola, Faruk Nome

**Affiliations:** † Department of Chemistry, Federal University of Santa Catarina, 88040-900 Florianópolis, SC, Brazil; ‡ Departamento de Química Fundamental, Instituto de Química, Universidade de São Paulo, 05508-000 São Paulo, SP, Brazil

## Abstract

Zwitterionic micelles and ionic liquids play an important role as organized media that can tune interfacial properties and modulate reaction environments in aqueous solutions. Understanding how these systems influence reaction kinetics is essential for elucidating catalytic mechanisms. This work investigates the catalytic effects of zwitterionic micelles on the decarboxylation of the 6-nitrobenzisoxazole-3-carboxylate anion (6-NBIC) and correlates kinetic parameters with the physicochemical properties of the self-assembled systems. Two classes of zwitterionic surfactants were examined: sulfobetaines (SB3-*n*) and an imidazolium-based surfactant (ImS3–14), the latter solubilized in aqueous solution by the ionic liquid BMIMBr. Critical micelle concentrations, interfacial properties, hydrodynamic diameters (4.7–7.7 nm), zeta potentials, and local micropolarity were determined. The micelles significantly enhanced reaction rates relative to aqueous solution, yielding intrinsic micelle/water rate ratios (*k*_M_/*k*_W_) of 376, 363, and 260 for SB3–14, SB3–14/BMIMBr, and ImS3–14/BMIMBr, respectively. Thermodynamic activation parameters in micellar systems revealed lower Δ*S* ^‡^ values than in aqueous solution, indicating more rigid transition states, along with reductions of approximately 10 kcal mol^–1^ in the activation energy. Specific ion effects at the micelle–water interface also modulate catalytic behavior by influencing zeta potential, ion–micelle interactions, and 6-NBIC/BMIM^+^ ion-pairing. These findings deepen the understanding of the mechanisms governing the catalytic effects of micellar systems in decarboxylation reactions.

## Graphical abstract

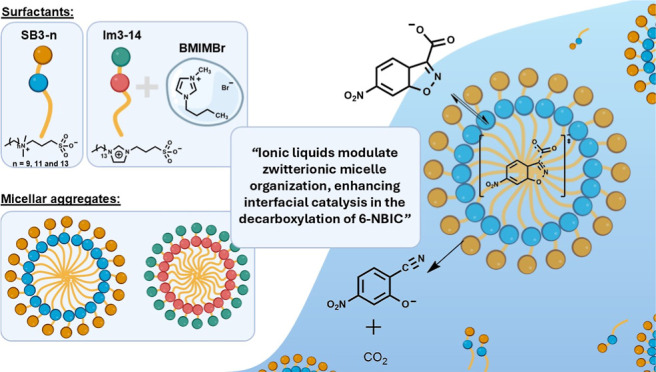

## 1 Introduction

Decarboxylation reactions have been investigated since the 19th century and continue to attract significant attention due to their central role in metabolic pathways and their synthetic relevance in organic chemistry.^1,2^ In aqueous solution, the efficiency of these reactions is strongly influenced by the medium’s polarity, the degree of solvation of the intermediates, and the stability of the transition state.^3^ Classical studies have shown that subtle adjustments in the dielectric constant can alter rate constants by several orders of magnitude, highlighting the sensitivity of this process to solvent–substrate interactions. Solvation effects are governed by the solvent’s polarity, the extent of delocalization of the anion formed upon dissociation, and the intrinsic rate of spontaneous decarboxylation.^3^

In this context, micellar systems have emerged as platforms capable of selectively modulating reaction microenvironments.^4–8^ Micelles form heterogeneous supramolecular domains^9^ that differ from bulk water in local micropolarity, organization, and charge density, thereby enabling control over reagent distribution, stabilization of charged species, and changes in activation thermodynamic parameters. These microenvironments, often described as “microreactors”, have demonstrated a strong ability to influence rates and mechanisms in a wide range of organic reactions.^10–13^

Among the different types of micelles, zwitterionic micelles stand out for exhibiting adaptive behavior, the so-called “chameleon effect”, which allows them to modulate their apparent charge in response to the nature and concentration of added electrolytes, the substrate type, or pH.^14,15^ This versatility brings these systems closer to the behavior observed in ionic liquids, expanding their potential for catalytic applications in aqueous media.^16^ These adaptive properties provide the system not only with highly dynamic behavior, but also with the ability to engage in selective interactions with both cationic and anionic species.^17^ In catalytic processes sensitive to charge distribution, such versatility becomes essential, as the simultaneous presence of polar and apolar domains within their structure resembles the behavior observed in ionic liquids, where organized regions can adapt and modulate electrostatic and hydrophobic interactions.^5,18^ The supramolecular organization and the generation of microenvironments with tunable polarity broaden the applicability of zwitterionic micelles, making them excellent platforms for micellar catalysis in aqueous media, particularly when selectivity control, polarity, and surface-charge adjustment, transition-state stabilization, and consequent increases in reaction rates are sought.^19,20^

Imidazolium-based ionic liquids have been widely reported to influence the interfacial behavior and micellization of surfactants through a combination of electrostatic and hydrophobic interactions. In particular, 1-butyl-3-methylimidazolium bromide (BMIMBr) has emerged as a representative model system due to the physicochemical characteristics of the BMIM^+^ cation. The imidazolium headgroup can interact electrostatically with polar moieties at the micellar interface, while the butyl side chain provides moderate hydrophobicity, promoting partial insertion into the interfacial region. Importantly, the C4 alkyl chain length offers a balance between hydrophilicity and hydrophobicity: shorter chains exhibit weaker hydrophobic interactions with micelles, whereas longer chains may induce self-aggregation or surfactant-like behavior. Consequently, BMIMBr allows the investigation of ionic liquid–micelle interactions without introducing significant independent aggregation.^21,22^ Previous studies have shown that such interactions can modify the critical micelle concentration, local polarity, and solvation of charged species, thereby stabilizing and reorganizing micellar interfaces. These features make BMIMBr particularly suitable for probing how ionic liquid-derived ions modulate supramolecular microenvironments and influence the catalytic behavior of micellar systems in aqueous media.^23–25^

In this work, we systematically investigate the influence of zwitterionic micelles formed by sulfobetaines (SB3-n) and an imidazolium-derived surfactant (ImS3–14) on the decarboxylation reaction of 6-NBIC. We evaluate how the ionic liquid BMIMBr modulates self-aggregation processes, interfacial properties, and, consequently, catalytic efficiency. Our findings demonstrate that structural adjustments within aggregates correlate strongly with kinetic and thermodynamic changes, revealing key principles for the rational design of micellar systems for catalytic applications in aqueous media.

## 2 Materials and Methods

### 2.1 Materials

The solvents used were all P.A., spectroscopic, or chromatographic grade. All solutions were prepared with Milli-Q ultrapure water. The aqueous solutions were prepared using BIS-TRIS buffer (Sigma-Aldrich), free of inorganic ions, at [BIS-TRIS] = 0.01 mol L^–1^ and pH 7.0 (HCl was used to adjust the pH, [Cl^–^] = 2.2 × 10^–3^ mol L^–1^). The fluorescent probe pyrene (purity ≥99.0%), and the surfactants SB3–10, SB3–12, and SB3–14 were purchased from Sigma-Aldrich. The surfactant ImS3–14 and the substrate 6-Nitro-benzisoxazol-3-carboxylate (6-NBIC) were synthesized and made available at the Laboratory of Catalysis and Interfacial Phenomena—LACFI. To control the pH of the solutions, a Metrohm model 713 potentiometer was used.

### 2.2 Synthesis of the Ionic Liquid 3-Butyl-1-methylimidazolium Bromide

The ionic liquid 3-Butyl-1-methylimidazolium bromide (BMIMBr) was synthesized through the bimolecular nucleophilic substitution reaction of butyl bromide and *N*-methylimidazole.^26^ The reaction was carried out in acetonitrile under reflux at 80 °C. Subsequently, the product was rotary evaporated and stored in a dry place. Scheme 1 shows the synthesis of BMIMBr.

**Scheme 1:**
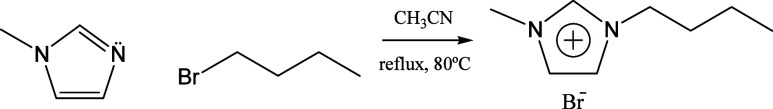
Synthesis of 1-Butyl-3-methylimidazolium Bromide

The product was characterized by ^1^H NMR (Figure S1) and mass spectrometry (Figure S2). Nuclear Magnetic Resonance (NMR) measurements were performed on a Bruker 200 MHz spectrometer. Tetramethylsilane (TMS, Cambridge Isotope Laboratories, 99.9%) was used as an internal reference for the analyses.

Mass spectrometric analysis of BMIMBr was performed using an Applied Biosystems 3200 QTRAP instrument equipped with an electrospray ionization (ESI) source operating in positive mode. The sample was dissolved in methanol at a concentration of approximately 10 μg mL^–1^ and directly infused into the ion source at a flow rate of 10 μL min^–1^. The source parameters were set as follows: ion spray voltage, 5500 V; source temperature, 400 °C; curtain gas, 20 psi; nebulizer gases GS1 and GS2, 40 psi; and declustering potential, 60 V. Full scan mass spectra were acquired over the *m*/*z* range of 50–500, using nitrogen as both the nebulizing and collision gas. Data acquisition and processing were performed using Analyst software (AB Sciex) and Origin9.

### 2.3 Methodology

#### 2.3.1 ImS3–14 Solubilization Using BMIMBr

The solubilization of ImS3–14 was monitored by resonance light scattering (RLS) using a Varian Cary Eclipse spectrofluorometer (synchronous mode with Δλ = 0) at different concentrations of the ionic liquid BMIMBr. The experiments were performed in a 0.01 mol L^–1^ BIS-TRIS buffer at pH 7.0 and 25 °C.

#### 2.3.2 Determination of the Hydrodynamic Diameter (*D*_h_) of Colloidal Systems

The hydrodynamic diameters of the micelles were obtained using a Brookhaven Zeta Plus/Bi-MAS dynamic light scattering device with a laser operating at 657 nm and a scattering angle of 90°. *D*_h_ values of the colloidal aggregates ([SB3-X] = 0.1 mol L^–1^, [ImS3–14] = 0.01 mol L^–1^, and [BMIMBr] = 0.025 mol L^–1^, when used) were determined in the presence of 0.01 mol L^–1^ BIS-TRIS buffer, pH 7.0, and 25.0 °C. The solutions were prepared and handled in a clean chamber (TROX fume hood, class 100). All samples were filtered through a 0.20 μm PVDF filter before analysis.

#### 2.3.3 Surface Tension Studies: Determination of Critical Micelle Concentration

The critical micelle concentration of the evaluated systems was determined from surface tension measurements at different surfactant concentrations, at pH 7.0 (BIS-TRIS Buffer, 0.01 mol L^–1^), and [BMIMBr] = 0.025 mol L^–1^ (when used). Surface tension measurements of the solutions were performed using a KRŰSS Easy Dyne interfacial tensiometer, model K8, equipped with the plate-and-ring method and coupled to a thermostatic bath at 25.0 °C.

#### 2.3.4 Determination of the Zeta Potential of Micellar Systems

The zeta potentials of the micellar systems were determined using Brookhaven’s dynamic light scattering (DLS ZetaPlus) system with the zeta potential analyzer. Measurements were performed in the presence of 0.01 mol L^–1^ BIS-TRIS buffer, pH 7.0, and 25.0 °C. The solutions of [ImS3–14] = 0.01 mol L^–1^ and [SB3–14] = 0.1 mol L^–1^ were prepared and handled in a clean chamber (TROX fume hood, class 100). All samples were filtered through a 0.20 μm PVDF filter before analysis.

#### 2.3.5 Polarity Studies of Colloidal Systems: Pyrene Polarity Scale

The local micropolarity of the SB3–14 and ImS3–14 micellar systems was probed via the fluorescence emission of pyrene, using the ratio between the emission bands I_I_ and I_III_ at 373 and 383 nm, respectively.^27–29^ It should be noted that the pyrene I_I_/I_III_ ratio reflects an average microenvironment sampled by the probe within the micellar interface rather than a single, well-defined interfacial polarity. The steady-state fluorescence emission measurements were performed using a Cary Eclipse spectrofluorometer (Varian). The spectra were used without correction. The pyrene concentration was 5 × 10^–7^ mol L^–1^, an appropriately low concentration to avoid internal filter problems and excimer formation. Measurements were carried out at 25 °C, 0.01 mol L^–1^ surfactant, and pH 7.0 (0.01 mol L^–1^ BIS-TRIS buffer).

#### 2.3.6 Kinetics of Decarboxylation Reactions of 6-Nitro-benzisoxazol-3-carboxylate (6-NBIC)

The kinetics were monitored by UV–vis absorption spectroscopy, using a Varian Cary 50 spectrophotometer coupled to a Peltier temperature control system. The reactions were carried out in quartz cuvettes with a total volume of 3.5 mL and a 1 cm optical path, containing 2 mL of solution. Aqueous solutions were prepared under the desired experimental conditions (SB3–14 or ImS3–14 concentration and BMIMBr concentration). These solutions were transferred to cuvettes, where the reactions were initiated by the addition of 10 μL of the 6-NBIC stock solution (0.01 mol L^–1^) in acetonitrile, yielding a final concentration of 5 × 10^–5^ mol L^–1^. Kinetic experiments were carried out at pH 7.0 (BIS-TRIS buffer, 0.01 mol L^–1^) at a controlled temperature and in triplicate.

To monitor the reactions, the kinetics were evaluated at the wavelength of maximum absorption of the band corresponding to the formation of the product 4-nitrosalicylonitrile (405–420 nm). The observed rate constants (*k*_obs_) were obtained by iterative first-order fits of the kinetics.

## 3 Results and Discussion

### 3.1 ImS3–14 Solubilization Using BMIMBr

The ImS3–14 surfactant is practically insoluble in water, forming a cloudy suspension indicative of large micrometer-scale aggregates. Nevertheless, this surfactant is known to form smaller micellar systems under specific conditions, such as elevated temperature or in the presence of ions.^30,31^ In the present study, the ionic liquid BMIMBr was employed to promote the solubilization of ImS3–14 in aqueous solution.

Resonance light scattering (RLS) spectra (Figure 1) revealed a pronounced decrease in scattering intensity with increasing BMIMBr concentration, consistent with a reduction in aggregate size.^32^ Above 0.0175 mol L^–1^ of BMIMBr, the solutions became completely transparent, confirming the disappearance of turbidity both visually and spectroscopically. These results demonstrate that BMIMBr effectively promotes the formation of smaller colloidal aggregates, yielding mixed assemblies of the zwitterionic surfactant and ionic liquid (ImS3–14/BMIMBr). For subsequent studies, a concentration of 0.025 mol L^–1^ BMIMBr was used to solubilize ImS3–14, ensuring that the BMIMBr concentration was relatively low but still well above the amount required for surfactant solubility.

**Figure 1:**
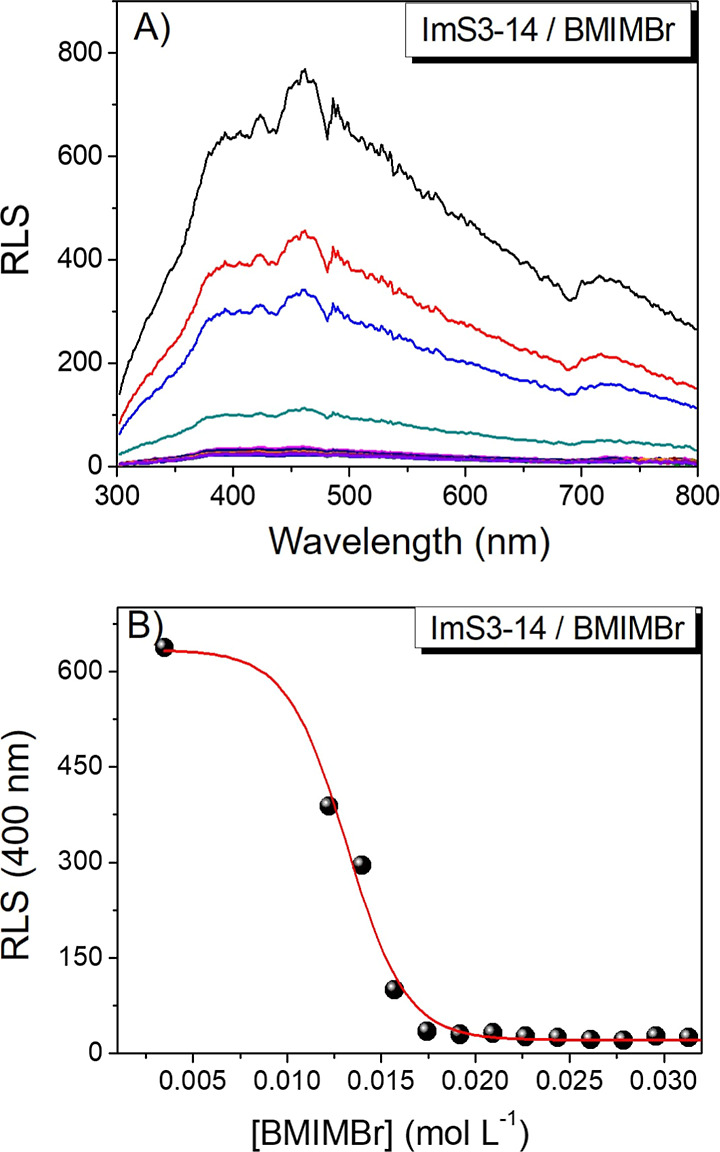
(A) Resonance light scattering (RLS) spectra and (B) RLS Intensity at 400 nm of ImS3–14 colloidal aggregates at different concentrations of the ionic liquid BMIMBr.

### 3.2 Determination of Critical Micelle Concentration, Hydrodynamic Size, and Surface Properties

The critical micelle concentrations (CMC) of the surfactants ImS3–14/BMIMBr, SB3–14, SB3–12, and SB3–10 in BIS-TRIS buffer were determined from surface tension measurements, as shown in Figure 2. As expected, the initial addition of surfactant molecules to the aqueous medium reduces surface tension through preferential adsorption at the air–water interface, whereas above the critical micelle concentration (CMC), the surface tension remains nearly constant with increasing concentration. The CMC values were obtained from the inflection points of the surface tension curves (Figure 2), and the results are summarized in Table 1. The surfactants ImS3–14 and SB3–14 have the lowest CMC values, as expected due to their longer hydrophobic chain length.^33^ Additionally, the CMC of ImS3–14 is lower than that of SB3–14, which may be ascribed to the presence of the imidazole moiety in the headgroup.^31^ The CMC values obtained in this study at pH 7 (BIS-TRIS buffer) are slightly lower than those reported in the literature at pH 9.06 (borate buffer) and can be attributed to differences in experimental conditions. In particular, the presence of Cl^–^ (2.2 × 10^–3^ mol L^–1^) in the buffer used here is expected to promote micellization, in agreement with previous findings.^34^

**Figure 2:**
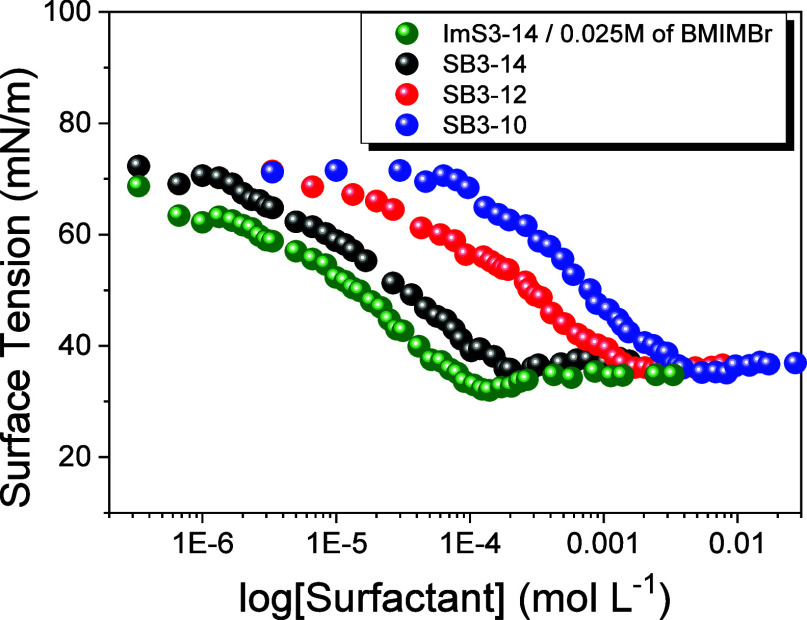
Surface tension dependence on surfactant concentration for determination of the critical micelle concentration at pH 7.0 (0.01 mol L^–1^ BIS-TRIS) and 25.0 °C.

**Table 1:** Critical Micelle Concentrations and Hydrodynamic Diameters of Zwitterionic Micellar Systems, at pH 7.0 and 25.0 °C^a^

surfactant	CMC (mol L^–1^)	CMC (mol L^–1^)^b^	*D*_h_ (nm)^c^	PDI
SB3–10	0.51 × 10^–2^	1.66 × 10^–2^	4.3	0.19
SB3–12	2.02 × 10^–3^	2.10 × 10^–3^	6.2	0.15
SB3–14	2.14 × 10^–4^	2.75 × 10^–4^	6.7	0.08
			5.8^a^	0.19
ImS3–14/BMIMBr (0.025 mol L^–1^)	1.12 × 10^–4^	--	7.7	0.22

a *D*_h_ of SB3–14 in the presence of 0.025 mol L^–1^ BMIMBr.

b Ref 35 at pH 9.06 in borate buffer.

c [SB3-X] = 0.1 mol L^–1^, [ImS3–14] = 0.01 mol L^–1^ and [BMIMBr] = 0.025 mol L^–1^.

The hydrodynamic diameters (*D*_h_) of the micellar systems were determined by dynamic light scattering for SB3–10, SB3–12, SB3–14, SB3–14/BMIMBr, and ImS3–14/BMIMBr, and the results are summarized in Table 1. All aggregates displayed nanometric *D*_h_ values, confirming their nature as nanostructured colloidal assemblies, with ImS3–14/BMIMBr > SB3–14 > SB3–12 > SB3–14/BMIMBr > SB3–10. Within the sulfobetaine series, micelles formed by surfactants with longer alkyl chains exhibited larger hydrodynamic diameters compared to those with shorter chains, following the order SB3–14 > SB3–12 > SB3–10. A slight decrease (∼1 nm) in *D*_h_ was observed for SB3–14 upon addition of 0.025 mol L^–1^ BMIMBr, consistent with previous work^36^ showing that the hydrodynamic diameter of SB3–14 micelles is sensitive to the type of ions present in solution. The effect of BMIMBr on ImS3–14 was much more pronounced, with the system evolving from a turbid suspension (Figure 1) to a transparent solution containing micelles of ∼ 7.7 nm. The polydispersity index (PDI) values ranged from 0.08 to 0.22, indicating a narrow size distribution and low polydispersity of the micellar aggregates formed in solution.

Surface tension measurements were used to determine interfacial properties.^37^ The maximum surface excess concentrations (Γ_max_) and the minimum areas per surfactant molecule (*A*_min_) at the air–liquid interface were obtained from the surface tension data using eqs 1 and 2, respectively.

1 $$\Gamma_{\max}=-\frac{1}{RT}{\left[\frac{\delta \gamma }{\delta \ln C}\right]}_{T,P}$$

2 $$A_{\max}=\frac{1}{N_{A}\Gamma_{\max}}$$

where *R* is the universal gas constant (8.314 J mol^–1^ K^–1^), *N*_a_ is Avogadro’s number (6.022 × 10^23^ mol^–1^), γ is the surface tension, and *C* is the surfactant concentration in solution. The value of the surface pressure at the CMC, Π_CMC_, was obtained using eq 3, where γ_0_ is the surface tension of the solvent and γ_CMC_ is the surface tension at the CMC. The values of Γ_max_, *A*_min,_ and Π_CMC_ are listed in Table S1.

3 $$\Pi_{CMC}=\gamma_{0}-\gamma_{CMC}$$

Despite the slight decrease in the CMC of ImS3–14 compared to SB3–14, both surfactants exhibited comparable surface properties, with similar Γ_max_, *A*_min_, and Π_CMC_ values (Table S1). Surfactants with longer hydrophobic chains exhibited higher Γ_max_ due to their greater tendency to adsorb at the interface, increasing the surface concentration.

### 3.3 Effect of BMIMBr on the Zeta Potential and Pyrene Polarity Scale of the Micellar Systems

To investigate in detail the effect of BMIMBr concentration on the micellar systems formed by ImS3–14 and SB3–14, zeta potential measurements were performed at varying concentrations of the ionic liquid. The zeta potential results obtained for ImS3–14 at different BMIMBr concentrations are shown in Figure 3A. The data reveal that the zeta potential initially becomes more negative (1), followed by a reversal in which the magnitude of the negative zeta potential progressively decreases as the BMIMBr concentration increases (2), before leveling off near its initial value. This trend indicates that two distinct processes occur as the ionic liquid concentration increases. The first stage (1), characterized by an increase in the magnitude of the negative zeta potential, can be attributed to preferential association of bromide anions with the surface of the ImS3–14 micelles. In the second stage (2), the progressively less negative zeta potential reflects the subsequent interaction of the BMIM^+^ cation with the micellar interface. This behavior is consistent with the well-documented ion-binding properties of zwitterionic micelles, in which more polarizable anions preferentially associate with the interfacial region before significant cation incorporation occurs.^38^

**Figure 3:**
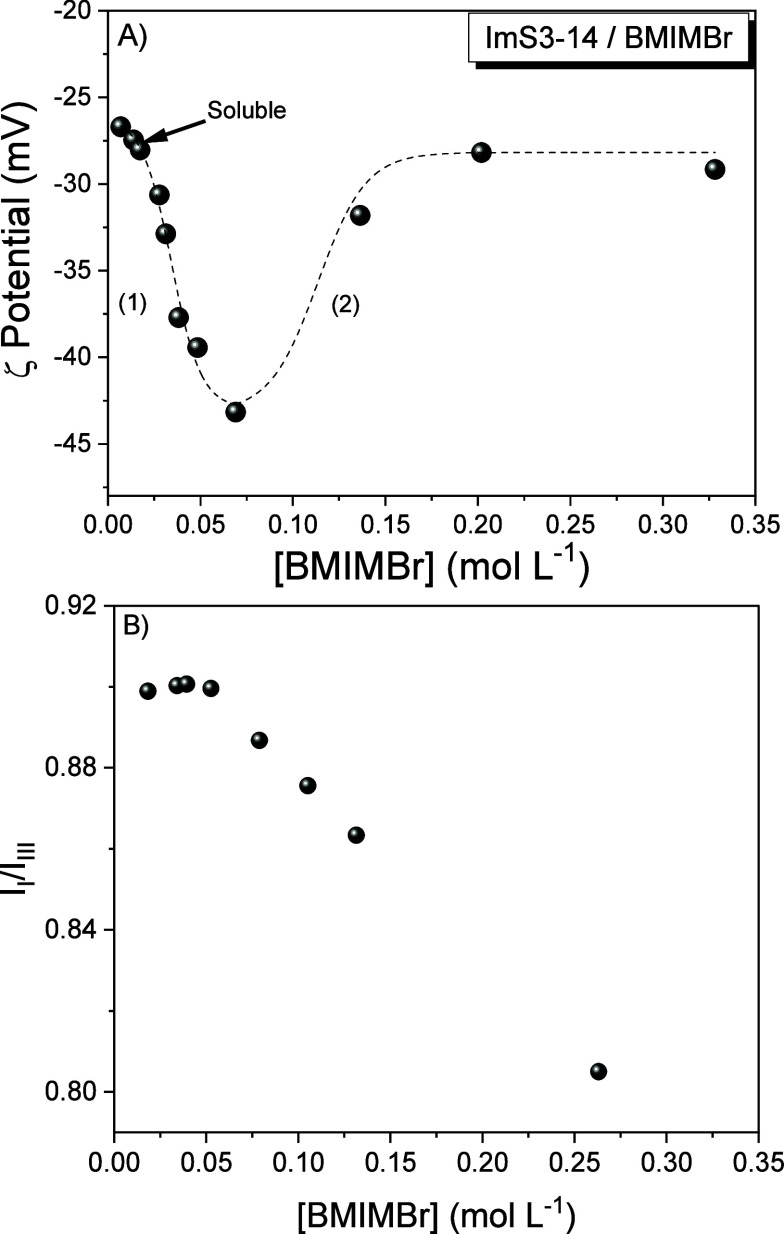
(A) Zeta potentials of the ImS3–14 micellar systems at different concentrations of the ionic liquid BMIMBr. (B) Ratio of fluorescence emission intensities of the vibronic bands I and III (I_I_/I_III_) of pyrene (5 × 10^–7^ mol L^–1^) at different concentrations of BMIMBr. Experiments were performed at pH 7.0, 25 °C, and [ImS3–14] = 0.01 mol L^–1^.

It should be noted that chloride ions are present in the system due to the BIS-TRIS/HCl buffer used for pH control and may also interact with the micellar interface. However, the chloride concentration remains constant throughout all experiments, whereas the concentrations of BMIM^+^ and Br^–^ increase progressively upon addition of BMIMBr. According to the Hofmeister series, Br^–^ generally exhibits a higher interfacial affinity than Cl^–^ owing to its greater polarizability and weaker hydration.^38^ Consequently, although competitive binding of Cl^–^ cannot be excluded, the progressive changes observed upon BMIMBr addition are most plausibly associated with the preferential incorporation of Br^–^ and BMIM^+^ at the micelle–water interface.

Moreover, although the increase in the magnitude of the negative ζ-potential was initially attributed to Br^–^ association at the micellar interface, specific ion binding in zwitterionic systems is often accompanied by structural reorganization. In sulfobetaine-type micelles, halide ions such as Br^–^ may influence headgroup hydration, dipole orientation, and Stern-layer organization, thereby modifying the interfacial electrostatic potential. Therefore, the observed change in ζ-potential likely reflects not only direct Br^–^ association but also ion-induced rearrangement of the interfacial structure.

To further evaluate the effect of BMIMBr on micellar systems, the zeta potential of SB3–14 micelles was measured at different BMIMBr concentrations (Figure S3). The data show an initial sharp increase in the magnitude of the negative zeta potential followed by a pronounced decrease in magnitude, eventually approaching values close to zero. This behavior is likewise attributed to initial Br^–^ binding to micelles, followed by BMIM^+^ binding, in agreement with isothermal titration calorimetric measurements of the micellization of SB3–14 in the presence of chloride, bromide, iodide, and perchlorate salts of BMIM^+^.^26^ Compared to ImS3–14 (Figure S4), SB3–14 exhibits a more pronounced variation, but in both cases the results clearly demonstrate that BMIMBr can effectively modulate the interfacial properties of zwitterionic micelles.

In addition, pyrene was employed as a fluorescent probe to assess differences in the local micropolarity of the zwitterionic micelles of SB3–14 and ImS3–14. Fluorescence emission spectra, recorded in the presence of varying concentrations of BMIMBr, were normalized to the intensity of the vibronic band III at 383 nm (I_I_). Representative spectra for ImS3–14/BMIMBr are shown in Figure S5, and the ratio of vibronic bands I (373 nm) and III, which reflects the pyrene polarity scale, is presented in Figure 3B. The I_I_/I_III_ ratios remained essentially constant up to the BMIMBr concentration corresponding to the point of transition between regions (1) and (2) in the zeta potential (Figure 3A), after which a progressive decrease was observed, indicating a reduction in the local micropolarity of ImS3–14. A similar analysis was performed for SB3–14 in the presence of BMIMBr (Figure S6). Analogous behavior was observed for the I_I_/I_III_ ratios of SB3–14 (Figure S6B). This two-stage profile is consistent with the specific binding of negative and positively charged ions discussed previously and suggests that bromide binding has little effect on the local interfacial micropolarity of the micellar system. In contrast, binding of the 1-butyl-3-methylimidazolium cation (BMIM^+^), which possesses a certain degree of hydrophobicity, leads to a measurable decrease in the microenvironmental polarity relative to the three SB3-n surfactants in the absence of BMIMBr (Figure S7). Similar results were reported for SB3–12 in the presence of low concentrations of BMIMBF_4_^34^ based on pyrene I_I_/I_III_ ratios.

### 3.4 Kinetics of the Decarboxylation Reaction of the 6-Nitro-benzisoxazol-3-carboxylate Anion

The unimolecular decarboxylation of the 6-nitrobenzisoxazole-3-carboxylate anion (6-NBIC) proceeds through delocalization of the carboxylate negative charge into the aromatic system, producing 6-nitrosalicylonitrile as product (Scheme 2). Consistent with previous reports, the reaction rate is negligible when the substrate is protonated (6-NBICH). Because the reported p*K*_a_ of 6-NBICH is 1.57,^39^ the substrate can be safely assumed to be in the fully unprotonated anionic form under all conditions at our experimental pH of 7 in the intermicellar aqueous phase.

**Scheme 2:**
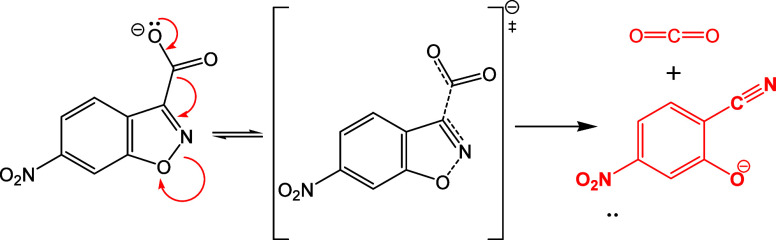
Decarboxylation Reaction of the 6-Nitro-Benzisoxazol-3-Carboxylate Anion (6-NBIC)

Decarboxylation kinetics were investigated in homogeneous buffer solution and in zwitterionic micelles composed of SB3–14, SB3–14/BMIMBr, and ImS3–14/BMIMBr, with reactions monitored by UV–Vis absorption spectroscopy at the product absorption band maximum at 418 nm (Figure S8A). Figure S8A shows the absorption spectral changes as a function of time for 6-NBIC decarboxylation in SB3–14/BMIMBr, with the growth of the product band at 418 nm. The isosbestic point at 345 nm indicates a clean conversion of the substrate into a single detectable product, consistent with the proposed mechanism.^39^ The kinetics of product formation at 418 nm and the corresponding fit of the data to a first-order kinetic model are shown in Figure S8B, from which observed rate constants (*k*_obs_) were obtained.

The decarboxylation reaction of 6-NBIC in ImS3–14, investigated over a range of BMIMBr concentrations, revealed two distinct kinetic profiles (Figure 4A). The observed decrease in *k*_obs_ with increasing BMIMBr exhibits a rather sharp change at a point (P_c_) that coincides with the reversal in the zeta potential measurements (Figure 4A) and with the more pronounced change in the local micropolarity sensed by pyrene fluorescence (Figure 4B). The initial reduction in *k*_obs_ can be attributed to the more negative zeta potential up to the point P_C_, which disfavors the binding of the anionic substrate 6-NBIC to the micellar interface. This interpretation is consistent with the fact that anionic micelles have only a limited catalytic effect on 6-NBIC decarboxylation.^40^ The micelle-excluded substrate in the aqueous phase experiences a more polar environment, which destabilizes the transition state while stabilizing the ground state, thereby lowering *k*_obs_. The post-P_c_ decrease in the magnitude of the negative zeta potential and the concomitant decrease in local micropolarity might lead one to expect an increase in the reaction rate at the higher BMIMBr concentrations. Instead, a progressive decrease in *k*_obs_ is observed as the BMIMBr concentration increases. Moreover, the decrease in *k*_obs_ correlates well with the increasing incorporation of BMIM^+^ into the micelles, as reflected in the changes in zeta potential (Figure 4). This behavior is consistent with ion-pair formation between the carboxylate anion of 6-NBIC and the BMIM^+^ cation at the ImS3–14 micelle–water interface, favored by their high local concentrations and the less polar interfacial environment. Such an electrostatic association can reduce the availability and reactivity of the carboxylate group toward decarboxylation. Although this effect differs mechanistically from protonation, which directly alters the electronic structure of the substrate, the formation of a tight ion pair can produce a similar inhibitory kinetic effect by stabilizing the carboxylate form and modifying its local solvation environment.

**Figure 4:**
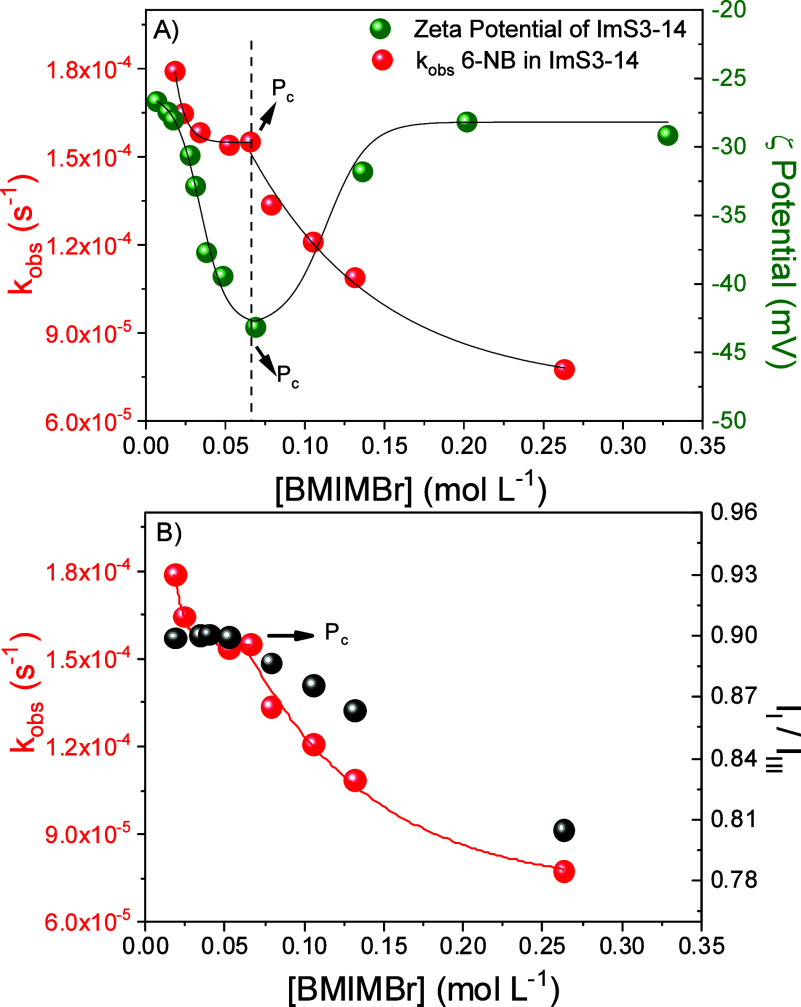
Rate constants of the decarboxylation reactions of 6-NBIC/ImS3–14 at different concentrations of BMIMBr compared to (A) the zeta potential and (B) the pyrene polarity scale. [6-NBIC] = 5 × 10^–5^ mol L^–1^, [ImS3–14] = 0.01 mol L^–1^, pH 7.00 and 25.0 °C.

#### 3.4.1 Evaluation of the Effect of Surfactant Concentration

Decarboxylation reactions of 6-NBIC were conducted in the presence of different concentrations of the surfactants SB3–14, SB3–14/BMIMBr, and ImS3–14/BMIMBr. Although BMIMBr is essential for the solubilization of ImS3–14, it reduces the rate of 6-NBIC decarboxylation at concentrations above P_c_ (Figure 4) and hence the catalytic effect of the micellar systems. For this reason, a moderately low BMIMBr concentration (0.025 mol L^–1^), well below *Pc* yet sufficient for good solubilization of ImS3–14 (Figure 1), was employed. Figure 5 presents the observed rate constants (*k*_obs_) for the reaction under each condition. Increasing the surfactant concentration led to a pronounced increase in *k*_obs_ values, approaching a plateau as the substrate was completely incorporated into the micellar pseudophase. To obtain the intrinsic first-order rate constant in the micellar pseudophase (*k*_M_), which represents the maximum reaction rate of the substrate in the micellar catalyst, the binding curves shown in Figure 5 were analyzed in terms of the appropriate pseudophase rate expression eq 4.^41^

4 $$k_{obs}=\frac{k_{w}+k_{M}K_{s}\left[Surfactant\right]}{\left(1+K_{s}\left[Surfactant\right]\right)}$$

**Figure 5:**
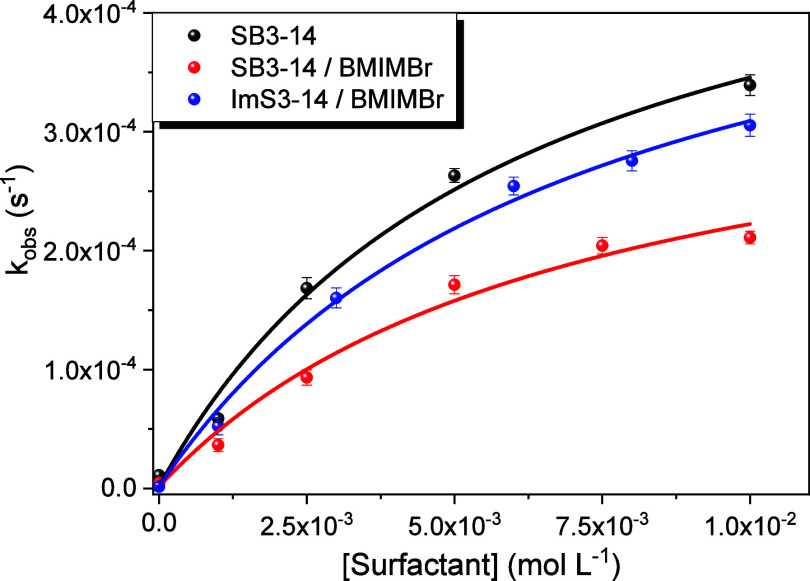
Effect of surfactant concentration on 6-NBIC decarboxylation reactions. [6-NBIC] = 5 × 10^–5^ mol L^–1^, [BMIMBr] = 0.025 mol L^–1^ (when indicated), at pH 7.0 and 25.0 °C.

The results obtained from fitting the substrate/micelle binding curves are presented in Table 2, including the substrate–micelle partitioning coefficient (*K*_S_), the rate constant in the micellar pseudophase (*k*_M_), and the ratio between the intrinsic first-order rate constants in the micellar and aqueous pseudophases (*k*_w_). The partitioning coefficients *K*_S_ for the interaction between the substrate and the micellar systems follow the trend SB3–14 > SB3–14/BMIMBr ≈ ImS3–14/BMIMBr, whereas the order of the *k*_M_/*k*_w_ values is SB3–14 ≈ SB3–14/BMIMBr > ImS3–14/BMIMBr. These findings highlight the importance of probe partitioning into the micellar interface in modulating the net catalytic effect.

**Table 2:** Substrate/Micelle Partitioning Coefficient, Micellar Phase Rate Constants, and Ratio between Micellar and Aqueous Phase Rate Constants^a^

colloidal catalyst system	*K*s mol^–1^ L	*k*_M_ ×10^–4^ s^–1^	*k*_M_/k_w_ ^b^
ImS3–14/BMIMBr	142.3 ± 8.5	3.8 ± 0.1	260
SB3–14/BMIMBr	140.0 ± 4.1	5.3 ± 0.2	363
SB3–14	165.4 ± 16	5.5 ± 0.1	376

a [6-NBIC] = 5 × 10^–5^ mol L^–1^, [BMIMBr] = 0.025 mol L^–1^, pH 7.0 and 25.0 °C.

b *k*_w_ = 1.46 × 10^–6^ s^–1^.

The *k*_M_/*k*_w_ ratios, which express the extent to which the reaction rate is enhanced in the micellar pseudophase relative to the aqueous medium, i.e., the net catalytic enhancement of the rate of the decarboxylation of 6-NBIC induced by these nanoreactors. Accordingly, the reaction in ImS3–14/BMIMBr exhibited a 260-fold increase in the rate constant, SB3–14/BMIMBr showed a 363-fold increase, and SB3–14 alone resulted in a 376-fold increase. All of these values are considerably higher than those observed for micelles of the analogous cationic surfactant CTABr, which exhibited only a 110-fold rate enhancement.^42^ This correlation indicates that zwitterionic micellar systems may be more promising candidates for catalyzing decarboxylation reactions of this nature.

#### 3.4.2 Thermodynamic Activation Parameters

The thermodynamic activation parameters for the decarboxylation of 6-NBIC were determined in aqueous buffer and in micellar solutions of SB3–14, SB3–14/BMIMBr, and ImS3–14/BMIMBr. The activation free energies (Δ*G* ^‡^) at 25.0 °C were calculated using eq 5.^43^

5 $${\Delta G}^{‡}=-RTln\left(\frac{k_{obs}h}{k_{B}T}\right)$$

where h is the Planck constant (6.626 × 10^–34^ J s), T is the absolute temperature in Kelvin, and *k*_B_ is the Boltzmann constant (1.381 × 10^–23^ J K^–1^).

To determine the activation enthalpies (Δ*H* ^‡^) for these reactions, kinetic measurements were carried out in the temperature range from 25 to 50 °C, and the resultant *k*_obs_ values plotted according to the Eyring equation (eq 6).^44^

6 $$\ln\left(\frac{k_{obs}}{T}\right)=\ln\left(\frac{k_{B}}{h}\right)+\frac{{\Delta S}^{‡}}{R}-\frac{{\Delta H}^{‡}}{R}\left(\frac{1}{T}\right)$$

as exemplified by Figure S9 for the decarboxylation of 6-NBIC in micellar SB3–14. From the known values of Δ*G* ^‡^ and Δ*H* ^‡^, the activation entropy (Δ*S* ^‡^) was determined using eq 7

7 $${\Delta G}^{‡}={\Delta H}^{‡}-T{\Delta S}^{‡}$$

and the activation energy (*E*_a_) using eq 8 ^44^

8 $$E_{a}={\Delta H}^{‡}+RT$$

The values of the thermodynamic activation parameters of the 6-NBIC decarboxylation reactions in aqueous buffer and in the different nanostructured zwitterionic micellar systems ([surfactant] = 0.010 mol L^–1^) are presented in Table 3. The activation parameters obtained in aqueous buffer are consistent with those reported in the literature, with experimentally determined values of Δ*H* ^‡^ = 34 kcal mol^–1^ and Δ*S* ^‡^ = 29 cal mol^–1^ K^–1^, compared with literature values of Δ*H* ^‡^ = 33 kcal mol^–1^ and Δ*S* ^‡^ = 27 cal mol^–1^ K^–1^.^42^ This agreement provides additional support for the reliability of the activation parameters determined in the presence of the micellar systems. A decrease in activation parameters relative to water is observed for the catalytic systems, i.e., in Δ*H* ^‡^, Δ*S* ^‡^, and *E*_a_. Furthermore, these values were quite similar for all the micellar systems, particularly between SB3–14/BMIMBr and SB3–14. This relationship indicates that, at this concentration, the ionic liquid BMIMBr has a relatively minor influence on the catalytic properties of the micellar systems. In addition, the similarity in the activation parameters of the catalytic systems indicates that all of them can be effectively employed in the catalysis of these reactions.

**Table 3:** Thermodynamic Activation Parameters for the Decarboxylation of 6-NBIC in Aqueous Buffer and in the Different Colloidal Systems^a^ ^,^ ^b^

	Δ*G* ^‡^ (kcal mol^–1^)	Δ*H* ^‡^ (kcal mol^–1^)	Δ*S* ^‡^ (cal mol^–1^ K^–1^)	*E*_a_ (kcal mol^–1^)
Buffer	25 ± 0.2	34 ± 0.2	29 ± 0.1	35 ± 0.2
ImS3–14/BMIMBr	23 ± 0.2	25 ± 0.2	7.6 ± 0.1	25 ± 0.2
SB3–14/BMIMBr	22 ± 0.2	24 ± 0.2	5.2 ± 0.1	24 ± 0.2
SB3–14	22 ± 0.2	24 ± 0.2	5.2 ± 0.1	24 ± 0.2

a [Surfactant] = 0.010 mol L^–1^ and [6-NBIC] = 5 × 10^–5^ mol L^–1^, at pH 7.0.

b Δ*G* ^‡^, Δ*S* ^‡^, and *E*_a_ calculated at 298.15 K (25.0 °C).

To elucidate the energetic effects on the transition state governing the decarboxylation of 6-NBIC, it is necessary to examine the parameters Δ*H* ^‡^, Δ*S* ^‡^, and *E*_a_. The activation energy and Δ*H* ^‡^ are essentially equivalent since the difference between them corresponds merely to RT (eq 7). Thus, the substantial reduction in the value of *E*_a_, on the order of approximately 10 kcal mol^–1^ in the presence of micellar systems, strongly reinforces the intrinsic catalytic effect of these systems.

The activation entropy, Δ*S* ^‡^, is highly relevant for mechanistic interpretation, as it reflects the degree of freedom of the transition state. For instance, the Δ*S* ^‡^ value in water is consistent with a unimolecular reaction that conserves charge,^39,42^ as proposed in the literature.^39^ On the other hand, the Δ*S* ^‡^ values for the reactions catalyzed by nanostructured micellar aggregates indicate a reduced degree of freedom in the transition state. In other words, the reaction proceeds through a more restrained transition state in the presence of the catalysts. This effect is likely due to interactions between the substrate and the more organized environment provided by the micellar aggregates, which restrict transition-state motions. The Δ*S* ^‡^ values obtained in the present work are consistent with those previously reported for the decarboxylation of 6-NBIC in other micellar systems,^42^ thereby reinforcing the reliability of the results.

## 4 Conclusions

The present study demonstrates that the ionic liquid BMIMBr can effectively modulate the self-assembly and interfacial behavior of zwitterionic surfactants, enabling the formation of well-defined, organized aggregates. The combined analysis of data for CMC, hydrodynamic size, zeta potential, and local micropolarity at the micelle–water interface reveals that BMIMBr induces distinct structural transitions via specific ionic interactions at the micelle–water interface, involving both Br^–^ and BMIM^+^. These effects highlight the sensitivity of zwitterionic micelles to ionic environments and the importance of ion-surfactant binding in determining their interfacial properties.

The evaluation of 6-NBIC decarboxylation shows that these micellar systems provide reaction environments that substantially enhance reaction rates. The reduced activation energy and a more constrained transition state indicate that the local structure and interfacial organization of the micellar systems play a central role in facilitating catalysis.

Taken together, the results offer detailed insights into how ionic liquid-derived electrolytes influence the colloidal behavior of zwitterionic micelles and how these structural modifications translate into catalytic improvements. These findings contribute to a broader understanding of ion-specific effects in micellar systems and support the rational design of self-assembled nanostructures with tunable interfacial and catalytic properties.

## Supplementary Material



## Data Availability

All data generated or analyzed during this study are included in this published article.

## Notes

The Supporting Information includes the structural characterization of BMIMBr by ^1^H NMR and mass spectrometry (Figures S1–S2), as well as the evaluation of its effect on zwitterionic micellar systems. Changes in interfacial properties were assessed by zeta potential measurements for SB3–14 and ImS3–14 aggregates (Figures S3–S4). The micellar microenvironment was investigated using pyrene fluorescence, with emission spectra and I_I_/I_III_ ratios indicating polarity variations upon BMIMBr addition (Figures S5–S7). The kinetics of 6-NBIC decarboxylation were monitored by UV–Vis spectroscopy and analyzed by monoexponential fitting (Figure S8), while activation parameters were obtained from the Eyring plot (Figure S9). Surface properties of the surfactants are summarized in Table S1 (PDF)
