# Supplementary material for: Ionic Liquid-Mediated Modulation of Zwitterionic Micelles and Their Catalytic Performance in the Decarboxylation of 6‑NBIC
Source: ACS Omega. 2026 Apr 27;11(18):27083–91. doi: 10.1021/acsomega.6c00719 (PMC13177019; doi:10.1021/acsomega.6c00719)
Supplement: Supplementary file 1 [file ao6c00719_si_001.pdf]

SUPPORTING INFORMATION

Ionic Liquid–Mediated Modulation of Zwitterionic Micelles and Their Catalytic Performance in the Decarboxylation of 6-NBIC

Paulo F. A. Costa,[a]* Victória R. Soares,[a] Yasmin S. Gomes,[a] Hugo Gallardo,[a] Frank H Quina,[b] Adriana P. Gerola,[a]* Faruk Nome[a,#]

[a] Department of Chemistry, Federal University of Santa Catarina, 88040-900 Florianópolis, SC, Brazil

[b] Departamento de Química Fundamental, Instituto de Química, Universidade de São Paulo, 05508-000 São Paulo, SP, Brazil

[#] *In Memoriam*

*Corresponding authors: pf.amaralcosta@gmail.com, adriana.gerola@ufsc.br

Address: R. Eng. Agronômico Andrei Cristian Ferreira, Zip Code 88040-900, Florianópolis, Santa Catarina, Brazil.

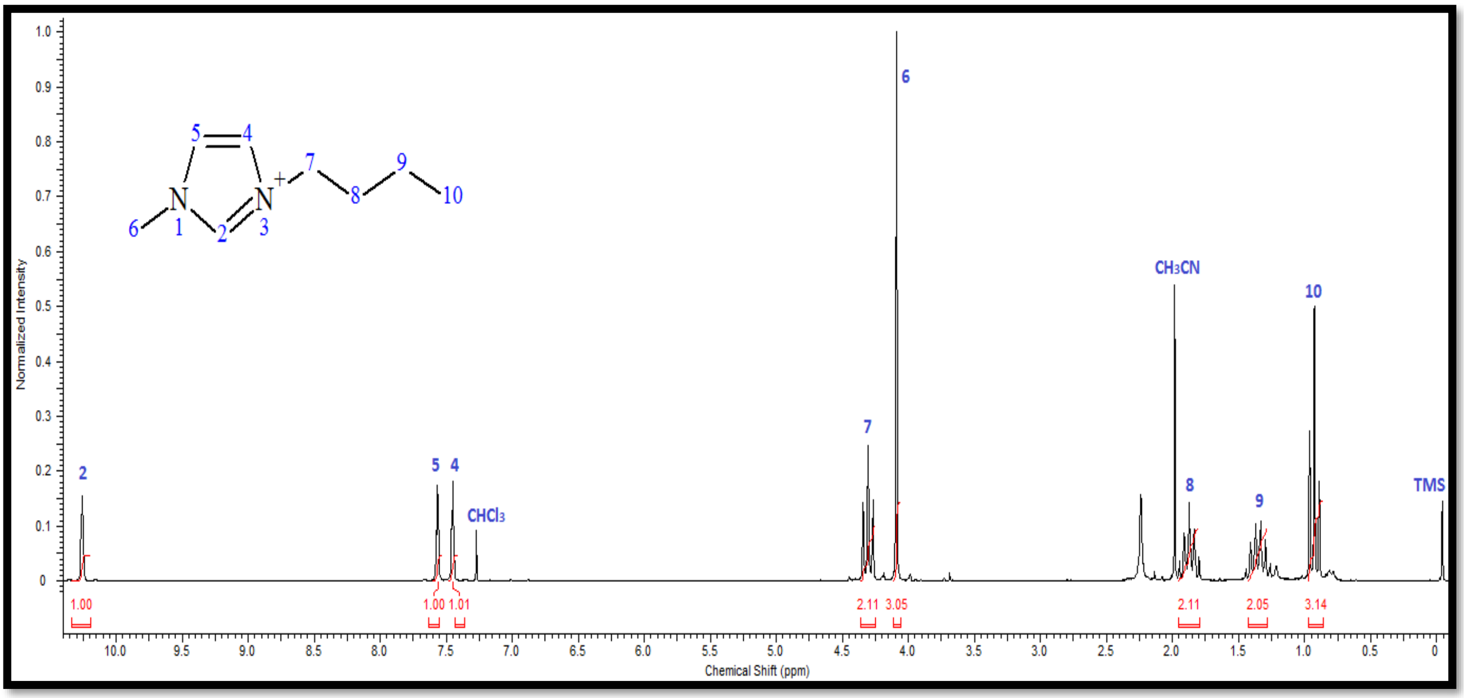

Figure S1. ^{1}H NMR of 1-butyl-3-methylimidazolium bromide. δ_H (200 MHz, $CDCl_3$): 10.30 (1H, s, 2); 7.61 (1H, s, 4); 7.49 (1H, s, 5); 4.35 (2H, t, 7); 4.13 (3H, s, 6); 1.92 (2H, qui, 8); 1.40 (2H, sex, 9); and 0.97 (3H, t, 10).

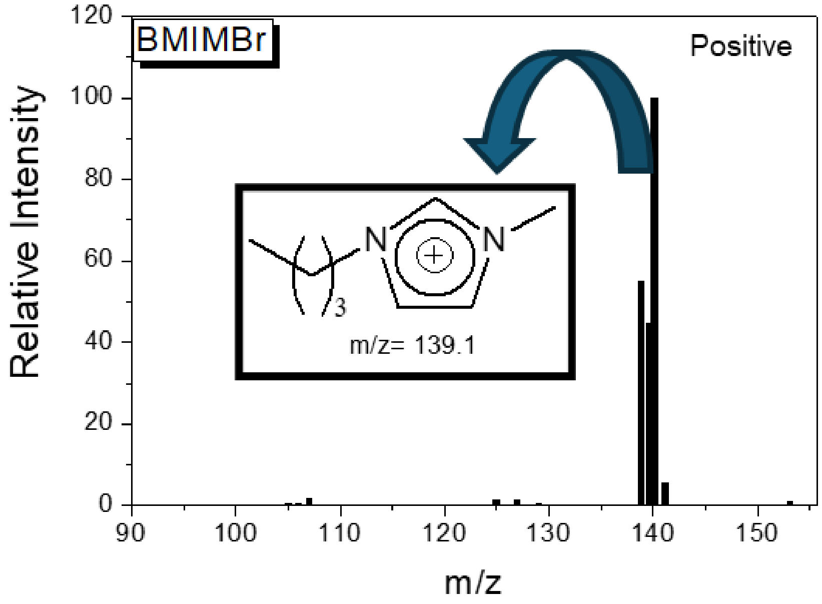

Figure S2. Mass spectrum for characterization of the ionic liquid BMIMBr in positive mode.

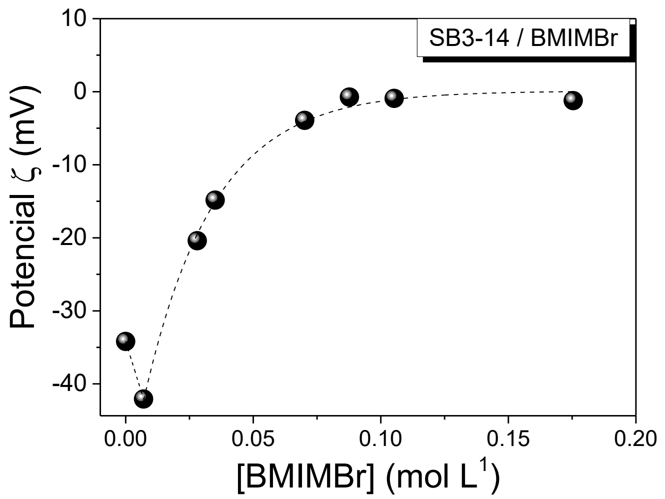

Figure S3. Zeta potentials of SB3-14 micellar aggregates at different concentrations of the ionic liquid BMIMBr at pH 7.0 and 25.0 ºC. [SB3-14]= 0.1 mol L^{-1}.

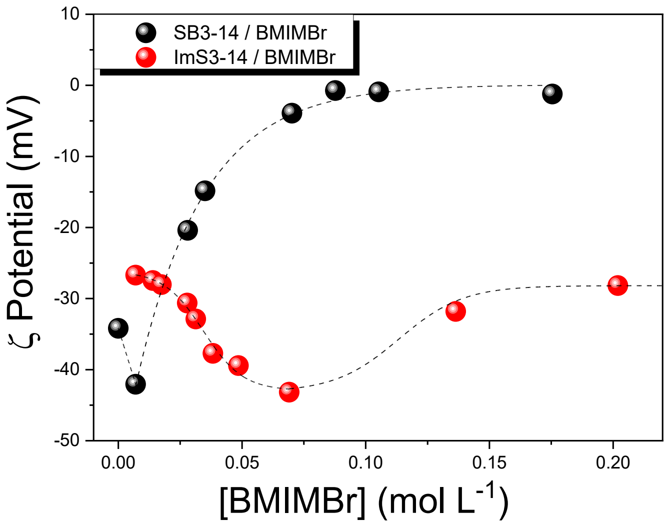

Figure S4. Superposition of zeta potentials of micellar aggregates of SB3-14 and ImS3-14 at different concentrations of the ionic liquid BMIMBr at pH 7.0 and 25.0 ºC. [SB3-14] = 0.1 mol L^{-1} and [ImS3-14] = 0.01 mol L^{-1}.

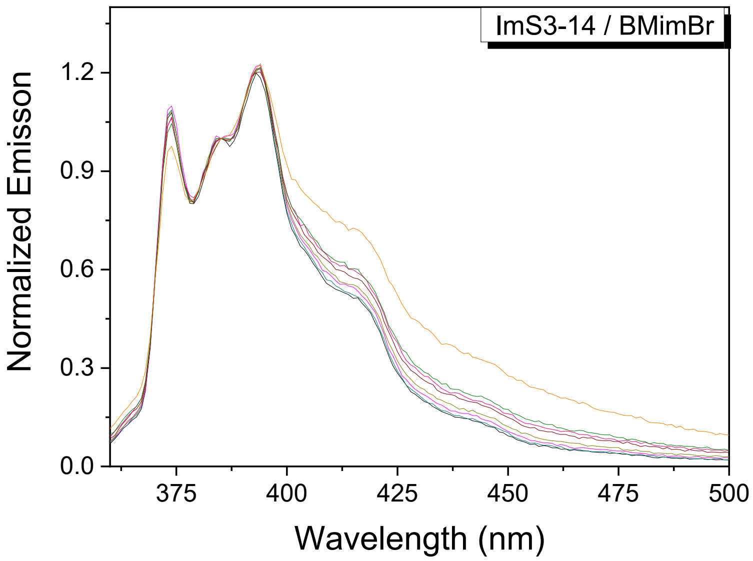

Figure S5. Emission spectrum of Pyrene (normalized to I_{III}) in 0.010 mol L^{-1} ImS3-14 at different concentrations of BMIMBr. [Pyrene]=$5x10^{-7}$ mol L^{-1}, pH 7.0 and 25 °C.

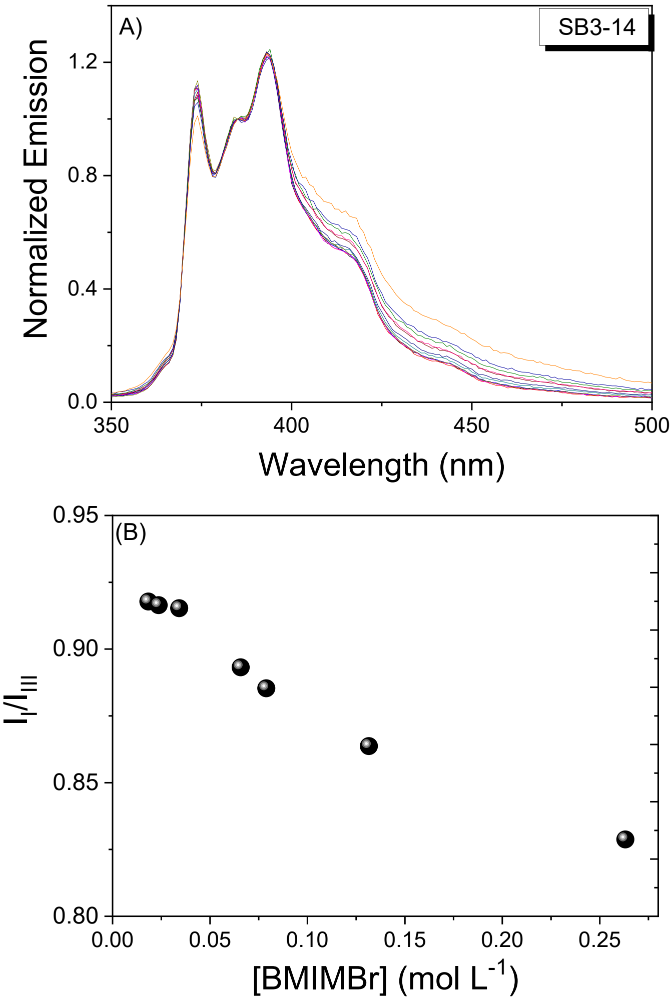

Figure S6. A) Emission spectrum of Pyrene (normalized to I_{III}) in SB3-14 (0.010 mol L^{-1}) and B) I_I/I_{III} ratio at different concentrations of BMIMBr. [Pyrene]=5x10^{-7} mol L^{-1}, pH=7.0 and 25 ºC.

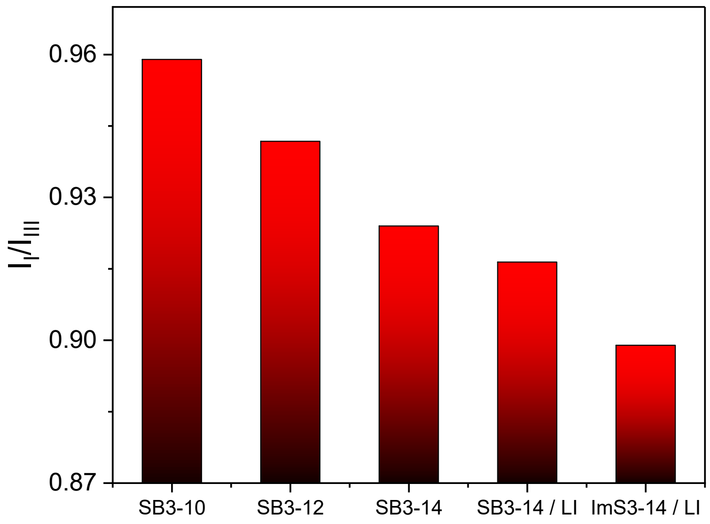

Figure S7. Relative pyrene polarities as reflected in the fluorescence vibronic band ratios (I_I/I_{III}) for the SB3-10, SB3-12, SB3-14, SB3-14/BMIMBr and ImS3-14/BMIMBr systems. [Pyrene]=5x10^{-7} mol L^{-1}, [BMIMBr]= 0.025 mol L^{-1} (when indicated as LI), at pH 7.0 and 25 °C.

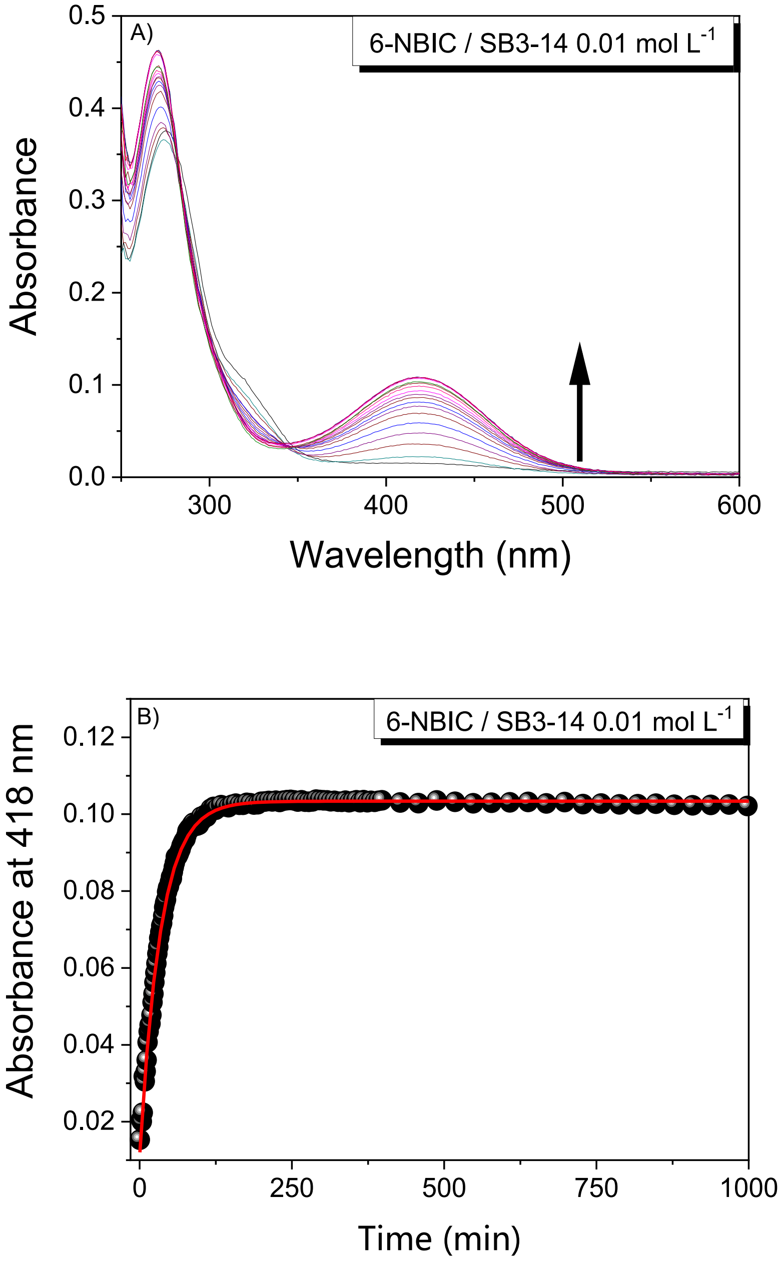

Figure S8. A) Kinetic monitoring of the decarboxylation reaction of 6-NBIC ($5x10^{-5}$ mol L^{-1}), at pH 7.0 and 25.0 ºC by UV-Vis spectroscopy B) Kinetic curve for the 6-NBIC decarboxylation reaction monitored by absorbance at 418 nm with a monoexponential growth fit. Formation of the product the 2-cyano-5-nitrophenolate anion.

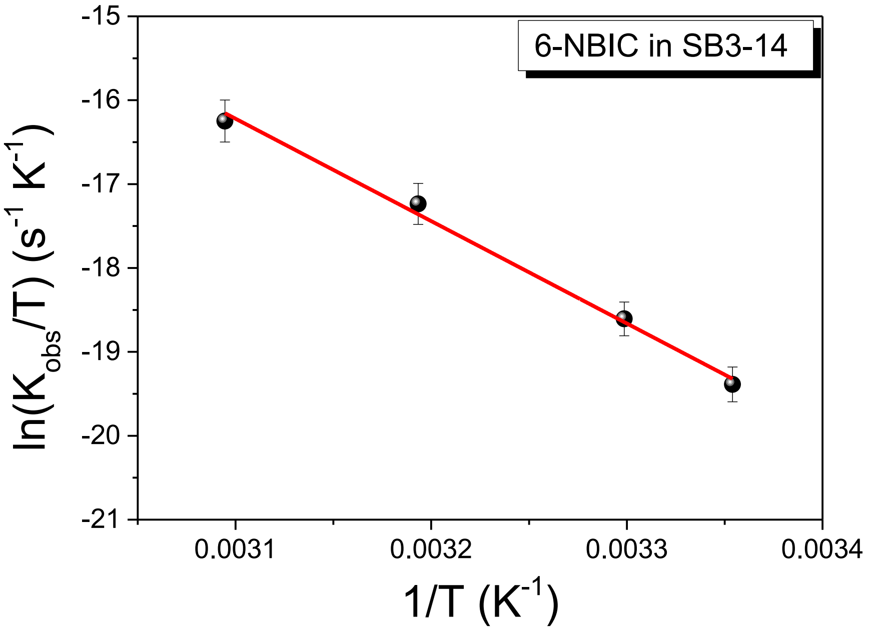

Figure S9. Eyring plot for the decarboxylation reaction of 6-NBIC in SB3-14. [6-NBIC] = 5x10^{-5} mol L^{-1}, [SB3-14] = 0.010 mol L^{-1}, at pH 7.0 and temperature from 25.0 to 50.0 ºC.

Table S1. Values of the maximum surface excess concentrations (Γ_{max}), minimum areas per surfactant molecule (A_{min}), and surface pressure at the cmc (Π_{cmc}) for the zwitterionic surfactants at pH 7.0 and 25.0 °C.

Surfactant	Π_{cmc} (mN/m)	Γ_{max} (10^{-6} mol/m^2)	A_{min} (Å^2)
**SB3-10**	36	3.0±0.1	56
**SB3-12**	36	2.6±0.1	63
**SB3-14**	36	3.3±0.1	51
**ImS3-14 / BMIMBr***	37	3.3±0.1	55

* [BMIMBr] = 0.025 mol L^{-1}
